# Puerperal Eclampsia

**Published:** 1912-02

**Authors:** J. S. Horseley

**Affiliations:** West Point, Ga.


### PUERPERAL ECLAMPSIA.

By J. S. Horseley, West Point, Ga.

Puerperal Eclampsia has been aptly called "The disease of theories." Thus announcing the unsatisfactory status of its etiology.

It would be an unnecessary consumption of your time to enumerate the various theories which have been advanced by different students in this field, accepted for a time, and then dropped because they were found to be unsatisfactory in meeting a sufficient number of the conditions of a working hypothesis.

The theory which has hindered progress more than any other in this study, is the association of uremia and albuminuria with this disease as cause. It is not difficult to see why this is so apt to be considered causal in the eclamptic seizure, since in such a great number of instances, it is present when the fit occurs, or appears very soon afterward. Few cases will be observed when the presence of albumin in the urine may not be easily demonstrated, and I might say that the greater the per cent. of albumin in the urine, the greater the danger to the life of the patient upon the supervention of eclampsia, so very naturally comes the idea of causal relation, and we might still continue to look in this field of investigation, if other conditions were not present bearing the same causal relation, viz., oedema, etc. But, since this is the main theory which is hindering observation, let us look into it for a while without prejudice and see if it is causal, or simply an accompanying condition.

1st. How and why does albumin appear in the urine of the pregnant woman—(I should say some pregnant women)? Let us suppose that the patient, previous to conception, was in a state of good health, as most of them are, and that having become pregnant, she in the later months develops eclampsia, and that albuminuria, et al., was present before, and at the time of the seizure. We will note that very soon after conception the patient has nausea and vomiting for some reason or other, the *probable* cause being disturbed digestion, with consequent irritation of products of decomposition upon the coats of the stomach. Changes in the pregnant uterus very easily disturb the nerves of the uterus, and this condition is referred to the stomach and its function is suspended. I believe, that if this particular condition (the disturbance of digestion, with its attendant results), is observed from the beginning of pregnancy to its ending, we will have eliminated eclampsia from the case.

At the beginning of pregnancy this function by its suspension or alteration, causes the appearance in the blood of toxins, or matters which are foreign to its normal constituents, and these toxins produce change (impairment or death) of the cellular elements concerned in function, everywhere, the kidney not excepted. Now, since the little tuft or capillary vessels contained within Bowman's capsule (the glomulerus), is the only part of the kidney where albumin can get into the urine (except where traumatisms occur); then let us see how this thing happens in the glomerulus.

All the blood going to the kidney for the purpose of function passes in through the renal artery, which, after division and subdivision, ends in the glomerulus as a tuft of capillary vessels. These in turn unite into a vessel (the efferent vein), which upon its emergence again subdivides into a capillary network around the convoluted tubes where the principal glandular work of the kidney is performed; these capillary vessels unite to form the renal vein. Now, albumin being a colloid body, cannot pass through an animal membrane, and since the basis membrane of the tubules is such, we must not expect albumin to pass the basis membrane of the capillary veins and the tubuli also. It follows that we must look for an easier way for it to get into the urine. We find this point of easier access in the glomerulus, where the only obstruction to its entrance is the capillary walls and cells, together with the epithelial cells lining the capsule and covering the glomerulus.

I refer you to works on anatomy for confirmation of the condition as to structure. As long as the circulating fluid is in a normal condition, the integrity of the capillary walls in the glomerulus and epithelial cells within the capsule can maintain their integrity, for they are doing just what they are designed to do; but when these toxins begin to appear in the blood sent to the kidney (as well as to other organs) the cells (endothelial) of the capillaries are damaged in the effort to excrete this poison, they lose their form, swell up, and after becoming thoroughly saturated, die, and the thin basis membrane becomes fissured by the force (the unexpected or unusual) of blood pressure which has been hitherto sustained by the endothelial cells. If the fissure is not too great only the serum of blood passes, if greater, blood also escapes into the glomerulus, and its cells also give way, and the serum passes into the beginning of the convoluted tube, albumin being one of its constituents, is found by the industrious doctor looking for the cause of eclampsia.

Please observe that processes similar to this one are going on everywhere in the whole body where we have a system of capillary vessels or functioning glands, which is simply repeating the same thing.

Note the swelling of the feet, legs, hands, face, etc. Simply endothelial cells unable to deal with their work and toxins added, fissures in the walls appear for the reason stated when speaking of the kidney, and the serum gets out of the vessels, into the interstitial parts, constituting dropsy or oedema, more or less general, according to the extent of the operating cause, which you will observe began with the disturbed function of digestion. For the same reason the cells of the liver swell up, rendering its efforts at function difficult (the patient becomes bilious). So with every part of the body as above stated, and it will be taking too much time to enumerate.

Now, gentlemen, I hope that I have shown you that there is a cause, lying behind the albuminuria, upon which *it* depends in common, and that we must look to this other and more remote cause for the condition we are trying to treat. I will not here undertake to define the toxin or toxins in question, but simply say that they result from imperfectly performed digestion with the fermentation present in such cases, and that these toxins account for all the departures from healthly function, which belong exclusively to the pregnant state. We have before stated that this disturbed function is, in some sort, caused by referred, or reflex conditions in the uterus. What these conditions are should not necessarily employ our time. Everybody knows that a pregnant woman gets sick. And among the professional "the nausea and vomiting of pregnancy" is often not one of the least troubles we have to deal with. Every woman does not have nausea, neither is every woman pregnant threatened with eclampsia. Just why this is so we leave with the remark that the stability of equilibrium of the nervous system varies according to temperamental states of individuals, and each individual case entrusted to the physician should be studied by itself.

Nausea and vomiting usually cease about the fourth month. Not so in these eclamptic cases; the digestive disturbances continue. By the fourth month the stomach has, so to speak, become accustomed to its burdens and ceases to make complaint, even though conditions have slowly but steadily grown worse. and by the seventh month the patient begins to notice that her shoes make dents in her feet and her rings fit too tight. She is not aware that she is sick, on the contrary, says that she is in fine health, only she can't eat enough to satisfy her appetite. These symptoms gradually grow worse as pregnancy advances, and toward the end of the term she begins to have peculiar symptoms about the head, which sometimes aches or feels giddy, hears peculiar noises and vision is not good, etc., etc. Now, while this is not intended to be a picture of every case, yet it will fit a large majority.

The first indication of going wrong in a few cases, is the seizure; but careful inquiry will reveal an overloaded stomach at the time of seizure, and it is reasonable to suppose that the attack could have been kept off by a timely emptying of the stomach.

Now as to how the attack comes on: Let us observe that the nervous system has been suffering in common with other parts of the body, and its readiness to take on abnormal action, promoted thereby. The nerve centres become more excitable, and when action begins, is more apt to pursue an unnatural course, as influenced by other centres in juxtaposition. We have seen that nausea and vomiting is the natural expression of an overloaded or irritated stomach, and we know that vomiting is presided over by a centre in the medulla in close juxtaposition to the other motor centres. It is easy to believe that sensory impressions sent from the stomach to the vomiting centre, which should produce emesis, might easily affect a larger area than its own centre; that is, its impression might be distributed and affect the general motor centres, and according to a law, only one centre may be in violent operation at the same time,—hence eclampsia instead of emesis. Note the manner of approach, the interval of recurrence, etc., how nearly it conforms to vomiting protracted from causes located in the stomach.

Agreeing to the general proposition, that the sum of evils present contribute to the resulting eclamptic attack, yet all these may be present in an extreme degree, and no seizure occur, if only there is absent some exciting cause for emesis. The prodromes of the eclamptic seizure, and of an attack of vomiting, are entirely similar, and I am led to believe that what purposed to be a vomiting spell is transformed into a general convulsion. This is made easily possible by the resulting state of unstable equilibrium into which the whole nervous system has been placed by the toxins in the blood.

I hope that I have been understood thus far, so that the fundamental ideas of causation can be considered in the following order:

1. Indigestion continuing through the *whole term* of pregnancy, is the "sine qua non" basis of the eclamptic condition and seizure.

2nd. That the renal lesions *are only a part* of the general disturbance, and *not* causative in its production.

3rd. That a careful study and treatment of the whole term of pregnancy on this idea will practically eliminate eclampsia, exceptional cases granted.

4th. That the principal value of albumin in the urine as a symptom, is that we can more easily thereby note the gravity of the situation, the danger being pari-pasus with the extent of injury to this function, other manifestations being more difficult to examine.

Many difficulties will be present to hinder the physician in his endeavor to carry out any plan of treatment. The patient will decline to be restricted as to diet, or to take medication before an extreme degree of toxemia has developed, on the idea that since she has *a good appetite,* she needs no doctoring, and the excessive hunger in a majority of cases will brook no interference with its gratification, until danger is apparent to her. Often it is the case that the first notice given the attendant, is when labor is near the condition extreme, then the doctor is fortunate if his directions are carried out and he can tide the patient over the danger. A case in point was treated by me a few weeks ago. Primipara, aged 20, colored, lived in Marietta, Ga., was brought to her father's, living near West Point, seventh month of pregnancy; oedema general, legs and feet swelled tightly, extending to abdomen; tongue white, headache, hears noises of birds singing, etc., semi-unconscious at intervals of an hour or two. Appetite ravenous, bowels inactive. Patient was given 5 grs. saccharated calomel, followed by tablespoonful Epsom salts, every four hours until bowels became quite active. No food allowed on first day of treatment; after this buttermilk every three hours, day and night. Swelling all gone on fourth day, head clear, feels well. Confined two weeks later of premature child, which died a few days later. No accidents or convulsions. Convalescence normal. I forgot to state that the urine became nearly solid upon boiling. Salts given every day after subsidence of swelling. I think this case would have had convulsions in a few hours under previous conditions. Clearing out bowels and keeping them so, with dieting, constitued treatment, except that 10 drops of chloroform was given in simple elixir four times a day after feeding.

To make the matter simple: give saline purgatives in sufficient quantity daily and diet to suit the individual case. While the rule is that patients will easily be relieved by the prophylactic treatment outlined, occasionally, eclampsia will occur after the subsidence of all the subjective symptoms, and the patient in apparently good health.

I must believe that in such cases, something has been taken into the stomach which induces nausea and an effort to vomit, which by reason of the unstable condition of the nerve centres in the medulla, is changed into a general convulsion. When eclampsia occurs, it requires to be treated. I will not undertake to outline a treatment for it, simply referring you to the best experience of authors. I personally rely upon morphine, veratrum, chloroform, active purgatives, diaphoresis, venesection; any one or more of above remedies as seems suitable to the case.

Induction of labor as a prophylactic measure I have not mentioned, because it will seldom be required if the doctor has been able to direct the treatment from the beginning. But when all measures have failed to relieve, then recourse may be had to this measure.

